# The Cost and Burden of the Residency Match in Emergency Medicine

**DOI:** 10.5811/westjem.2016.10.31277

**Published:** 2016-12-19

**Authors:** Aaron M. Blackshaw, Simon C. Watson, Jeffrey S. Bush

**Affiliations:** The Medical University of South Carolina, Department of Medicine, Division of Emergency Medicine, Charleston, South Carolina

## Abstract

**Introduction:**

To obtain a residency match, medical students entering emergency medicine (EM) must complete away rotations, submit a number of lengthy applications, and travel to multiple programs to interview. The expenses incurred acquiring this residency position are burdensome, but there is little specialty-specific data estimating it. We sought to quantify the actual cost spent by medical students applying to EM residency programs by surveying students as they attended a residency interview.

**Methods:**

Researchers created a 16-item survey, which asked about the time and monetary costs associated with the entire EM residency application process. Applicants chosen to interview for an EM residency position at our institution were invited to complete the survey during their interview day.

**Results:**

In total, 66 out of a possible 81 residency applicants (an 81% response rate) completed our survey. The “average applicant” who interviewed at our residency program for the 2015–16 cycle completed 1.6 away, or “audition,” rotations, each costing an average of $1,065 to complete. This “average applicant” applied to 42.8 programs, and then attended 13.7 interviews. The cost of interviewing at our program averaged $342 and in *total*, an average of $8,312 would be spent in the pursuit of an EM residency.

**Conclusion:**

Due to multiple factors, the costs of securing an EM residency spot can be expensive. By understanding the components that are driving this trend, we hope that the academic EM community can explore avenues to help curtail these costs.

## INTRODUCTION

According to the American Association of Medical Colleges (AAMC) the median indebtedness for a U.S, medical school graduate in the class of 2015 was $183,000.^1^ Although the cost of tuition at institutions is obviously a tremendous burden for medical students, the expenses incurred acquiring a residency position also represent a problem for many students. For those going into the field of emergency medicine (EM) this cost can be especially onerous.

In the 2016 National Resident Matching Program (NRMP), there were 1,895 positions offered by 174 EM programs across the country. This cycle, an estimated 2,476 candidates (1,693 U.S. seniors) applied for these positions.^2^ When comparing NRMP applicant survey data from 2008 and 2015 it is clear that current medical students are submitting more applications (28.7 vs 39, ERAS costs of $351 vs $601 at current rates), attending more interviews (11.4 vs 13), and ranking more programs (10.7 vs 13) in order to match.^3^,^4^ This is perhaps partially explained by the risk and consequences of not matching, with a 99.9% match rate for EM in the standard match. Unsurprisingly, applicants are thus preparing and planning for the process with even more fervor.

One area stressed by applicants is obtaining strong standardized letters of evaluations (SLOEs). The SLOE was developed by the Council of Emergency Medicine Residency Directors (CORD) to provide a global perspective on an applicant’s candidacy by providing meaningful comparisons to peers applying to EM and it has also been proven to have impressive interrater reliability.^5^,^6^ Program directors of EM programs recently reported that the top three factors in deciding which applicants to invite for an interview were SLOEs, grades from EM rotations, and the United States Medical Licensing Examination (USMLE) Step 1 scores.^7^ In order to obtain more SLOEs, hopeful medical students are encouraged to complete at *least* one away rotation, but more commonly two, in addition to their home EM rotation.

Attending these away or “audition” rotations is obviously expensive, often requiring travel to and lodging in a new place for four weeks at a time. The application process through the Visiting Student Application Service (VSAS) is time consuming and also charges per application submitted.^8^ In fact, a cursory search of the VSAS EM elective catalog for the 2015–16 cycle reveals that 63% (114/155) of rotations have an additional institutional processing or application fee.^9^

Few data exist in the literature about the student-incurred costs of the fourth-year medical student during the final stages of their medical school training, including total number of residency interviews and number of away (audition) rotations. Two surveys administered to students seeking various residency positions in 1977 and 1986 reported the average costs incurred by students attending residency interviews to be $2,802 and $2,390 respectively (costs adjusted for inflation).^10^,^11^,^12^ Another study conducted on the 2006 urology match revealed the median total cost of the interview process to be $4,725, with a median per-interview cost of $390 (costs again adjusted for inflation).^13^

A large study of fourth-year medical students from 20 U.S. allopathic schools, which included 109 EM applicants from the 2013–14 cycle, showed that 89% completed an away rotation at great personal cost. Additionally, over 43% of the EM applicants spent more than $4,000 on the interview trail.^14^ A similar questionnaire administered by the AAMC to 59 EM hopefuls from the 2014–15 cycle revealed the average expenses incurred on the interview trail to be $3,936, although this only represented the cost of transportation and lodging.^15^

In this study, we sought to investigate *both* the financial and time burden that VSAS and the Electronic Residency Application Service (ERAS) applications represent, as well as the financial burden incurred by participating in away rotations and attending residency interviews. We focused on obtaining a better estimation of the *total* costs incurred by EM applicants during the process of matching into their desired specialty. Secondarily, we desired to gain a better understanding of the expenses incurred by interviewees at our program.

## METHODS

The authors created a 16-item survey that asked about both the time and monetary costs of the entire EM residency application process. The questionnaire was developed and subsequently edited to be pertinent, appropriate, and easily completed in just a few minutes. By design, no identifying information of the candidate was asked or recorded. A combination of multiple-choice and free-response questions was ultimately decided upon. Revisions of the survey were agreed upon by both the associate residency director and clerkship director of the program prior to administering the questionnaire.

The first six items on the survey were program specific to EM at our institution. Questions were directly related to travel and costs incurred by interviewees at our institution – a three-year residency at a quaternary care academic center in a medium-sized city located in the Southeastern United States. The rest were more generalized questions about the entire application and interview process.

All medical school applicants chosen to interview for an EM residency positions at our institution were invited to complete the survey during their interview day. Participation was completely voluntary, and no incentives for participation were offered. Applicants were notified that filling out the survey would have absolutely no bearing on their application, the rest of their interview day, or the ranking process.

The questionnaire was administered on paper, and then collected and securely stored by the program coordinator. After completion of *all* residency interviews for the 2016 cycle, we analyzed the entire collection of responses. All surveys were assigned random ID numbers and a database of all responses was created. Uncertainties uncovered during abstraction were resolved via group consensus. We converted any values reported as ranges to the exact midpoint for calculation purposes. Our institutional review board granted a waiver for this study.

## RESULTS

In total, 66 out of a possible 81 residency applicants (an 81% response rate) partially or fully completed a survey. Sixty-one of these candidates completed the institution-specific questions, items related specifically to our unique location. It was found that roughly 54% of applicants arrived for our interview by air with the remaining 46% traveling by car. Most students chose to stay at a local hotel (67%), followed by staying with friends and family (28%), and then bed and breakfasts (5%). Our invitation email, which included information about local lodging options was the most-used method (39% of respondents) for finding lodging. Hotel chain websites (26%), friends/family (26%), and AirBnB (8%) represented the other sources of finding a place to stay. The total cost of attending an interview at our program averaged $342, median cost was $325, with an interquartile range (IQR) of $185–500. Almost 60% (36/61) of applicants spent $200 or less on transportation to our program. A breakdown of lodging costs revealed that 61% of students paid $100 or less, and the rest paid between $101–200.

The remaining portion of the questionnaire related to applicant’s costs and experience with other, non-institutional specific aspects of their application process. Students were asked about multiple areas of monetary and time burdens as summarized in Table 1 and Table 2. We found that fourth-year students applied to an average of 4.4 away rotations and ultimately completed 1.6 of these. Completing VSAS took 20 hours or less for 88% of respondents.

The total cost of each away rotation was estimated to be $1,065, and a breakdown of this total cost revealed the most expensive portion of the process to be lodging ($526), followed by transportation at $251 (Figure).

Fourth-year medical students were advised to apply to an average of 38.5 residency programs and chose to actually apply to 42.8. This application process was more arduous compared to completing VSAS, with 64% of students taking longer than 20 hours to complete ERAS. On average, students were planning on attending 13.7 residency interviews and each one would cost applicants an average of $414. Transportation accounted for an average of $226 of this total cost, with lodging next at $122 (Figure).

The final question of the survey was open ended and asked applicants about other costs not listed on the questionnaire. The most commonly listed cost was that of buying interview attire and paying for dry cleaning. Others mentioned using airline miles to pay for tickets, as well as the indirect cost that many miles of travel would have on the wear and tear of their automobiles.

## DISCUSSION

The absolutely “average” applicant that interviewed at our residency program for the 2015–16 cycle would have applied to 4.4 programs through VSAS and subsequently completed 1.59 away rotations. VSAS fees assessed would total $86. These rotations would cost an average of $1,704 to complete. This student would then take a great deal of time to complete ERAS, applying to 42.8 programs, totaling $700 in ERAS fees. Additional costs for submitting the USMLE transcript ($80) and to register for the NRMP ($70) would also be assessed.^16^^,17^ Our average applicant would then attend 13.7 interviews at a cost of $414 per program. In *total*, this average applicant would have spent $8,312 in his or her pursuit of an EM residency. In context, the amount for residency application represents an approximate 4.4 % of the average medical school debt incurred.

## LIMITATIONS

Limitations of this study include the small number of respondents since we only included those students applying to our program, an academic center in a medium-sized city located in the Southeastern United States. Because the medical center is not in a large city, the cost to get here may be higher since the airport is not a major hub. We would expect the cost of travel to be less if there were a denser distribution of EM programs in this region of country. For example, travel distances between programs in the New England area or other large metropolitan area would be significantly less. Furthermore, the costs reported by the medical students are not all inclusive as the recollections of the survey respondents are probably less than 100% accurate and subjective.

### Future Directions

By understanding the costs of applying to residency programs, we hope to encourage discussions about ways to decrease the financial burden on future emergency physicians. Performing interviews online and helping to standardize the application process may be ways to accomplish this.

## CONCLUSION

In today’s economy, it is almost understood that going into the medical field means that the young physician will most likely incur debt due to medical school and postgraduate training. However, limited research has been done in looking at the expenses incurred while acquiring a residency position. Due to the increase in popularity of EM, current medical students entering the field are submitting more applications, attending more interviews, and ranking more programs in order to match. Furthermore, in order to obtain the SLOEs needed to make a student more competitive, they are being encouraged to complete away rotations, which add to their expenditures, and the cost of the application process through VSAS continues to climb. Due to all of these factors, the costs of securing an EM residency spot are escalating at an alarming rate. By understanding the components that are driving this trend, we hope that the academic EM community can explore avenues to help curtail these costs.

## Figures and Tables

**Figure:**
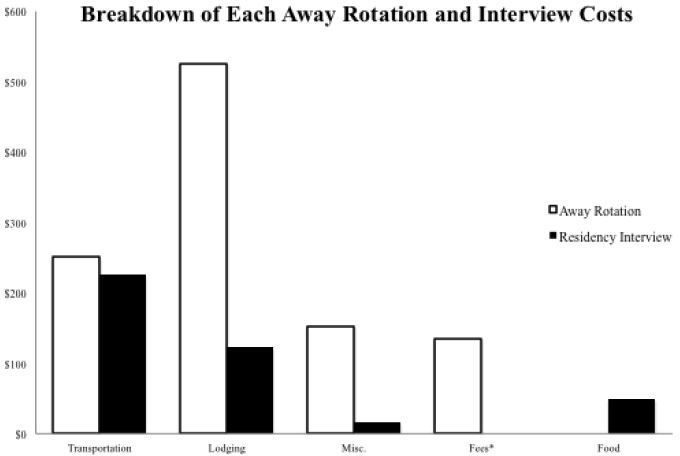
Breakdown of each away rotation and residency interview costs: Applicants were asked to breakdown the total costs of completing away rotations and attending residency interviews into individual components. ^*^Away rotations often charge fees that can include VSAS application fees, additional individual school fees, vaccination or titer requirements, drug screens, and malpractice insurance. *VSAS,* visiting student application service.

**Table 1:** Selected fourth-year medical students survey response data regarding costs incurred for away rotations and application to residency.

	No. of responses	Mean (SD)	Median (IQR)
No. of away rotations applied to	66 (100%)	4.4 (2.8)	4 (2–5)
No. of away rotations completed	66 (100%)	1.6 (0.7)	2 (1–2)
Total cost of each away rotation	63 (96%)	$1,065 (818)	$900 (490–1400)
No. of residency programs advised to apply to	64 (97%)	38.5 (13.1)	37.5 (30–41.3)
No. of residency programs applied to via ERAS	65 (98%)	42.8 (14)	40 (32.5–40)
No. of interviews applicants plan to attend	64 (97%)	13.7 (3.2)	13 (11.8–15.5)
Total cost of each interview	63 (96%)	$414 (167)	$410 (260–520)

*SD,* standard deviation; *IQR,* interquartile range; *ERAS,* electronic residency application service

**Table 2:** Total time spent completing VSAS and ERAS by medical students.

VSAS		ERAS	
	
Time (hrs)	No. of Responses (%)	Time (hrs)	No. of Responses (%)
0–10	35 (54)	0–20	23 (35)
10–20	22 (34)	20–40	28 (43)
20–30	5 (8)	40–60	10 (15)
>30	3 (5)	>60	4 (6)

*VSAS,* visiting student application service; *ERAS,* electronic residency application service
